# The Little-Known Ribbon-Shaped Piriform Cortex: A Key Node in Temporal Lobe Epilepsy—Anatomical Insights and Its Potential for Surgical Treatment

**DOI:** 10.3390/diagnostics14242838

**Published:** 2024-12-17

**Authors:** Pamela Ochoa-Lantigua, Jhinson Moreira-Mendoza, Cecilia Alejandra García Ríos, Jose A. Rodas, Jose E. Leon-Rojas

**Affiliations:** 1 NeurALL Research Group, Quito 170157, Ecuador; 2 Facultad Ciencias de la Salud, Universidad Nacional de Chimborazo (UNACH), Riobamba 060108, Ecuador; 3 School of Psychology, University College Dublin, D04 V1W8 Dublin, Ireland; 4 Escuela de Psicología, Universidad Espíritu Santo, Samborondón 092301, Ecuador; 5 Medical School, Universidad de las Américas (UDLA), Quito 170124, Ecuador

**Keywords:** piriform cortex, epilepsy, temporal lobe, LITT, epileptogenesis, drug resistance

## Abstract

The piriform cortex (PC) plays a pivotal role in the onset and propagation of temporal lobe epilepsy (TLE), making it a potential target for therapeutic interventions. This review delves into the anatomy and epileptogenic connections of the PC, highlighting its significance in seizure initiation and resistance to pharmacological treatments. Despite its importance, the PC remains underexplored in surgical approaches for TLE. We examine the specific neuroanatomy of the PC as well as the limitations of current imaging techniques and surgical interventions, emphasizing the need for improved imaging protocols to safely target the PC, especially in minimally invasive procedures. Furthermore, the PC’s proximity to vital structures, such as the lenticulostriate arteries, presents challenges that must be addressed in future research. By developing multimodal imaging techniques and refining surgical strategies, the PC could emerge as a crucial node in improving seizure freedom outcomes for TLE patients.

## 1. Introduction

Epilepsy is defined by the International League Against Epilepsy (ILAE) as a disease that includes at least two unprovoked seizures happening > 24 h apart, one unprovoked seizure with a likelihood of recurrence greater than 60% in 10 years, or an established diagnosis of an epilepsy syndrome [1]. Multiple etiologies have been reported and range from genetic factors to brain anatomical abnormalities [1,2]. It is known that approximately 50 million people in the world suffer from this disease [3].

Temporal Lobe Epilepsy (TLE) is the most common of the focal onset epilepsies and represents 40% of all cases in adults [1,2,4]. In such cases, the usual culprit is the Mesial Temporal Lobe, a complex anatomical region involving several structures, such as the hippocampus, parahippocampal gyrus, amygdala, entorhinal cortex, perirhinal cortex, and the piriform cortex (PC) [4]. TLE is strongly related to treatment resistance, especially when associated with Hippocampal Sclerosis (HS), which is the most common pathological finding in TLE patients [4,5,6]. When epilepsy is treated with two or more appropriate drug regimens, either combined or as monotherapy, and seizure freedom is not achieved, the patients are considered to have drug-resistant (DR) epilepsy [4,7]. In such patients, surgical intervention can be curative or significantly reduce the burden of DR epilepsy by achieving a seizure freedom rate of 58% at 1 year [8,9,10,11].

Even though multiple studies have shown the benefit of epilepsy surgery in patients with DR-TLE, around 20–30% who undergo this procedure persist with seizures after the first surgery and 53% after a repeat surgical intervention [12,13]. In a cohort study conducted in 2019, the role of PC resection was evaluated as a possible explanation for the lack of seizure freedom [14]. The results showed that patients in the seizure-free group had a higher proportion of the PC volume resected and that extirpation of more than 50% increased the odds of seizure freedom by sixteen-fold [14].

To further understand the role of the PC in seizure genesis and post-surgical outcome, this literature review has the objective of describing the PC and its role, beginning with its relationships with other brain structures and concluding with a description of the imaging techniques that can be used for its identification in the operating theater.

## 2. Biological Influence of the Piriform Cortex in Epileptogenesis

### 2.1. Anatomy of the Piriform Cortex

**Figure 1:**
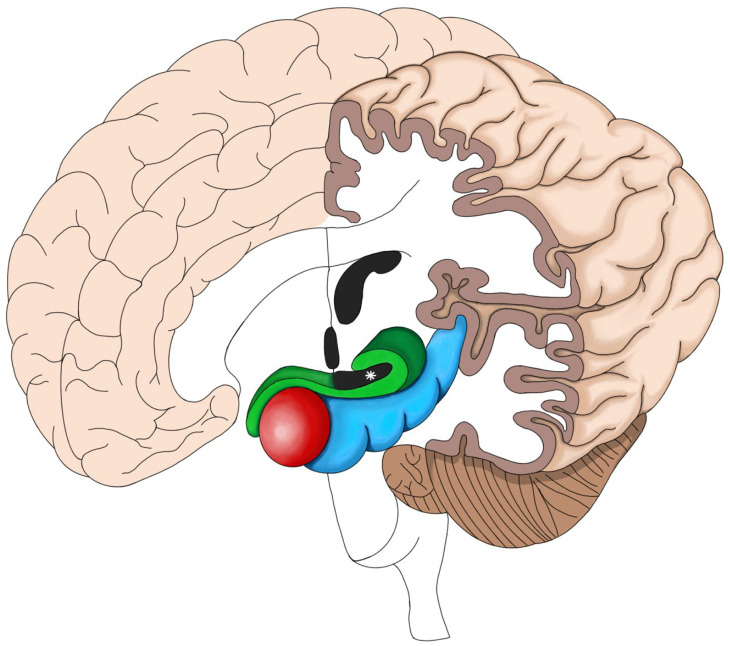
3D representation of the piriform cortex. Note the PC, in green, overlaying both the amygdala anteriorly (in red) and the hippocampus posteriorly (in blue). Also note the characteristic ribbon shape of the PC surrounding the entorhinal sulcus (ERS) (*).

**Figure 2:**
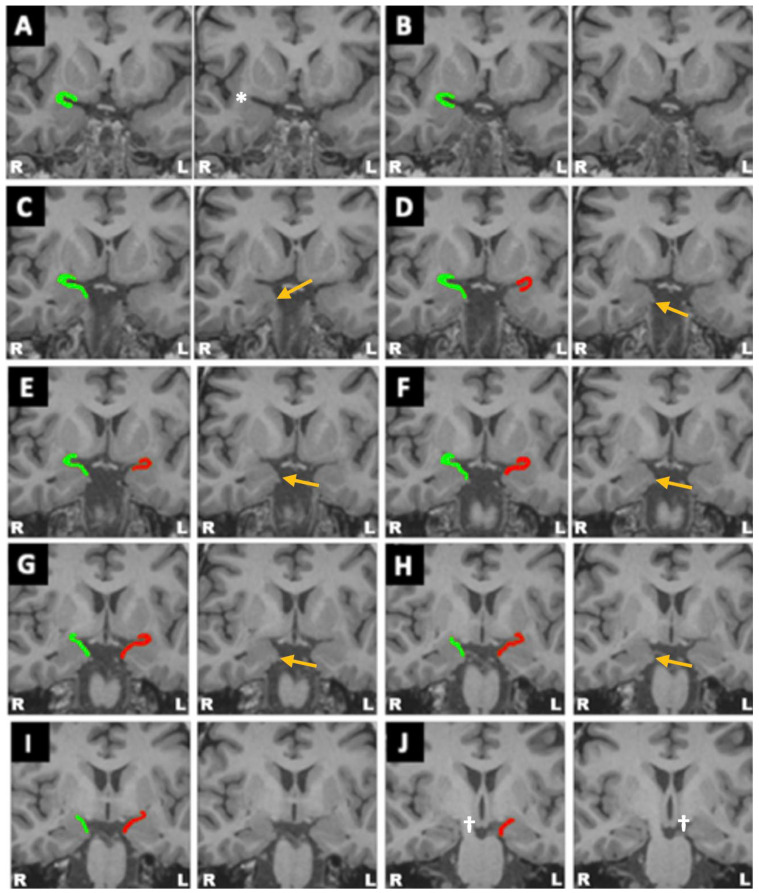
High-resolution MRI showcasing the PC and the relevant anatomical landmarks used to identify it; the right PC is highlighted in green, and the left PC is highlighted in red. Panels (**A**–**J**) showcase different coronal planes of showcasing the piriform from its most anterior aspect (**A**) to its most posterior aspect (**J**). The orange arrow shows the apex of the gyrus semilunaris (the lower limit of the PC), and the * shows the limen insulae, which marks the beginning of the PC (its anterior limit), while the appearance of the cerebral peduncles, labeled by the cross sign (†), marks the end of the PC (its posterior limit). Taken and modified from a previously published work belonging to the main author under a CC BY 4.0 license [18].

**Figure 3:**
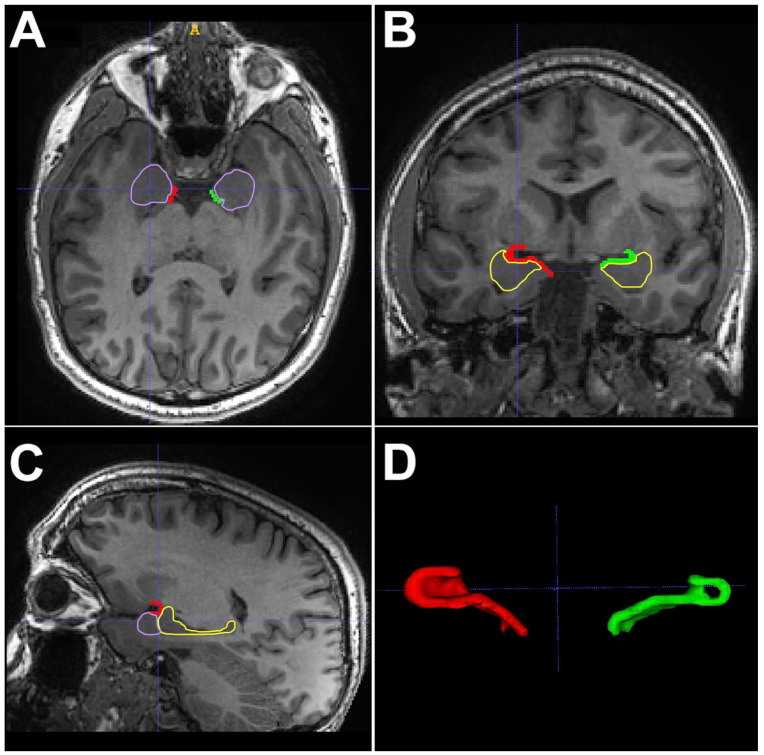
T1-weighted 3D MRI of the brain demonstrating the location of the PC and its relationships with the amygdala (purple) and hippocampus (yellow) in the axial (**panel A**), coronal (**panel B**), and sagittal (**panel C**) planes. Additionally, (**panel D**) shows a 3D reconstruction of the PC where the classic ribbon shape can be better appreciated.

Knowledge of the PC’s anatomy and neuronal connections is important for understanding the role it plays in TLE (Figure 1, Figure 2 and Figure 3). The PC is contained within the olfactory cortex; it embraces the superior and inferior banks of the entorhinal sulcus (ERS), which divides the PC into frontal (superior) and temporal (inferior) portions [15,16,17,18,19] (Figure 1, Figure 2 and Figure 3). The ERS is responsible for the characteristic “ribbon shape” of the PC [15,16]. Its location in the olfactory cortex can be the explanation for the olfactory auras, a phenomenon that occurs in 0.9–8.1% of focal TLE [14,15]. There is no clear consensus in the literature regarding the PC anatomical limits in humans as there is in animals, perhaps because it is not easily discernible macroscopically from the rest of the amygdala [14,15,16,17,20]. However, some anatomical landmarks can be useful for its delimitation. For example, its lateral aspect is bordered by the temporal stem, and its anterior limit is represented by the limen insulae (LI), while its posterior limit is represented by the appearance of the cerebral peduncles [15,16,17]. The PC lies over the amygdaloid nuclei anteriorly and the hippocampus posteriorly [15,17] (Figure 1). The latter is an anatomical structure that has played a central role in focal epilepsy research [15]. In addition to having a close anatomical relationship with the PC, the hippocampus has a similar histological structure in terms of neuronal organization, which might help explain the association between PC and focal epilepsies [15,21]. Lateral to the most prominent part of the PC, which borders the fundus of the ERS, a group of cells located in the Claustrum have been considered as the fourth layer of the PC and together form the “prepiriform claustrum” [15,16,20,22]. Another anatomical landmark is a small depression located in the medial temporal lobe, known as the sulcus semiannularis, which marks the posterior and inferior limits of the PC [15,17]. Finally, another landmark is the most prominent medial part of the gyrus semilunaris; the PC extends inferiorly until its apex (Figure 2). The PC has been the subject of multiple scientific studies and a focus of researchers who aim to elucidate its epileptogenic role through multiple means, one of which involves the induction of seizures in animal models in a process known as kindling [23].

### 2.2. Epileptogenesis and the Piriform Cortex

The complex architecture of the PC and its interconnections has been a subject of study in cellular and animal epilepsy models [21,24,25]. The most common model is the kindling model, which utilizes a series of periodic electrical stimulations to generate secondary generalized seizures, as well as spontaneous seizures and an epileptic state [23,26]. This model enables electrical sensitivity to persist for months, which permits the study of chronic brain dysfunctions [26]. However, long-term kindling models are a complex endeavor due to damage or misplacement of chronic implants, along with the high cost and amount of time spent in these procedures [23]. Nevertheless, the combination of the kindling model with EEG aids in the identification of the source of aberrant electrical discharges and its connections to other brain structures [27]. For example, a series of EEGs performed while kindling the limbic lobe showed a close interconnection between this area, the PC, and the motor system [28]. Other afferences that connect with the PC come from the entorhinal cortex, the cortical amygdala, the thalamus and hypothalamus, and different neuronal types arising from the brainstem (such as noradrenergic, serotonergic, cholinergic, and dopaminergic neurons) [15,21].

**Figure 4:**
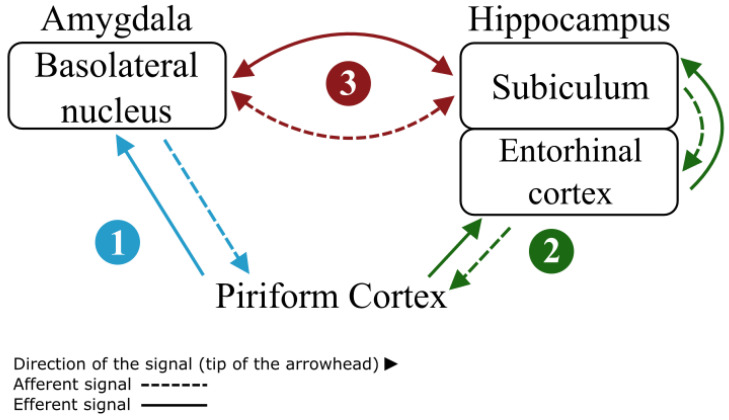
PC circuitry responsible for its epileptogenicity. Two main circuits connect the PC with relevant structures in the mesial temporal lobe: number (1) showcases the PC/Basolateral Nucleus of the Amygdala Circuit and number (2) shows the PC/Entorhinal Cortex Circuit. Finally, a feedback loop is created between these structures shown by number (3), which results in the reverberation of the epileptic activity as seen in TLE.

In addition to determining the PC’s neuronal connections, kindling has also identified it as an area responsible for seizure genesis, contributing to the initiation, propagation and generalization of the aberrant electrical stimulus [27]. There are many explanations as to why the PC acts as a generator node for TLE. First, studies have shown that GABA depletion causes the worsening of seizures, playing a relevant role in ictogenesis. Interestingly, the PC’s third layer has a GABAergic feedback inhibition hub, which increases neuronal excitability in this layer [21,29]. Second, other studies have shown that immediate-early genes (IEGs), indicators of neuronal activation such as c-fos and arc, are expressed early in the PC, within the first electrical discharge during kindling of the amygdala [30,31,32]. In contrast, in the hippocampus, the expression of these indicators requires longer discharges and more stimulations, demonstrating the higher sensitivity of the PC for seizure induction [30,31,32]. The expression of these IEGs stimulates neuronal plasticity which further increases susceptibility to long-term seizures [30,31,32]. Third, the circuits that connect with the PC confer it a potential for seizure generation and propagation, particularly the PC/Basolateral Nucleus of the Amygdala and the PC/Entorhinal Cortex Circuits [15,26,33], as seen in Figure 4. The latter connects the PC with the hippocampus and subiculum, two structures that can easily disseminate seizures due to their outputs to multiple brain regions [15,26,33]. These structures also have the potential to create a feedback loop, because they are connected afferently and efferently between each other, augmenting the epileptic activity in a process known as loop-gain amplification (Figure 4) [33].

Besides the PC’s susceptibility to electrical kindling, its neuronal circuits are also susceptible to seizure induction with chemoconvulsants [26,34,35]. Karen Gale, in 1988, described an area in the ventral aspect of the PC that was susceptible to chemoconvulsants (NMDA, glutamate, aspartate, and bicuculline), and that was suppressed by the injection of GABA agonists; she named it “area tempestas” [15,36]. Afterward, in 1995, Löscher and collaborators reported that an area in the rostral posterior PC within the third cell layer, corresponding to the pre-piriform claustrum in humans, showed more susceptibility to seizure generation and a significantly lower threshold for focal seizure genesis [20].

Finally, the PC is a principal olfactory area, essential for the processing and perception of odors; it amalgamates olfactory sensory inputs with superior cerebral regions, facilitating odor recognition, memory retention, and emotional reactions [21]. The participation of the PC in olfaction is significantly associated with its robust connections to other limbic regions, including the amygdala and hippocampus, as seen in Figure 4. These links indicate a common mechanism that may underlie the association between olfactory stimuli and TLE, as mentioned before [15,26,33]. Recent research suggests that olfactory cues may serve as triggers for seizures, especially in individuals with TLE. This occurrence, known as olfactory-induced epilepsy, may arise from the PC’s function as a seizure-initiating zone [21,22,24,33]. Abnormal hyperactivity in the piriform cortex following olfactory stimulation may transmit epileptiform activity to other areas in the mesial temporal lobe, facilitating the onset of seizures [33,34,35]. These findings highlight the necessity for additional research into the causal association between olfactory stimuli and epilepsy, and whether this relationship could be utilized for diagnostic or therapeutic applications. This new perspective underscores the dual function of the PC in olfaction and epileptogenesis, accentuating its clinical relevance in TLE.

Considering all the aforementioned factors regarding its epileptogenic potential, we can conclude that the PC might be a target for therapeutic interventions. Lesions of the PC have been associated with a reduction or halt of secondary generalization of focal seizures with a positive correlation between the extent of the lesion and the number of stimulations needed to reach a high-stage seizure (r = 0.66, *p* = 0.0055) [26,37]. Furthermore, in animal models, deep brain stimulation has resulted in a decreased after-discharge duration and a delay in seizure progression while targeting the central PC [15,38]. However, human application is difficult because of the uncertainty of its anatomical limits and the associated atrophy caused by longstanding TLE [38]. To develop novel epilepsy interventions that target the PC, a precise definition of its location is necessary through imaging techniques and algorithms.

## 3. The Role of Imaging in Epilepsy and Piriform Cortex Identification

Magnetic Resonance Imaging (MRI) has not only changed the way that epilepsy is diagnosed but has also aided in the determination of prognosis and treatment for patients with this disease [39]. The physical properties used for image generation and qualitative analysis also allow for the assessment of the brain’s general structure and microstructure with high-resolution imaging [40] and enable the identification of small lesions such as cortical malformations that might otherwise be missed [41]. In addition to this qualitative analysis, MRI provides a quantitative analysis of the intensity of signal within a single voxel or the diffusion of water molecules [42,43]. A useful MRI analysis used to detect volumetric differences within brain areas is calculated using Voxel-Based Morphometry (VBM), in which several images are compared with a template in order to detect changes that even expert human examiners could have normally missed [44,45]. For example, a case-control study assessing the changes in grey matter volume in TLE-HS patients using VBM, despite its small sample size, showed a significant decrease of grey matter volumes in mesial temporal structures ipsilateral to the TLE-HS [45]. These numerous small anatomical modifications in the brain signify a considerable deviation from typical anatomy and cannot be recognized through visual evaluation of MRI, sometimes being overlooked even by seasoned neuroradiologists. The construction of white and grey matter templates through VBM has facilitated the advancement of automatic segmentation algorithms that assist in identifying concealed epileptogenic lesions [46,47].

Certainly, the ability to precisely analyze the volumetry of different brain regions has improved epilepsy research, particularly thanks to the volumetric studies that have been performed in the hippocampus, which showed significant morphological changes induced by epilepsy [48,49,50]. In 1992, the first study that showcased the benefits and usefulness of volumetric studies was published; it specifically showed the usefulness of manual segmentation and region of interest volumetry (ROI) [48]. The hippocampus was segmented in a group of 40 patients diagnosed with epilepsy (20 with TLE and 20 with Frontal Lobe Epilepsy [FLE]) and 10 controls [48]. The imaging findings showed that in patients with TLE, the hippocampal volume ipsilateral to seizure onset had an asymmetric volume loss. The usefulness of these volumetry-based findings was not only exemplified by witnessing the lateralization of hippocampal atrophy but also by the differentiation of TLE from FLE, as neither the control group nor the FLE patients had hippocampal volume loss [48]. Therefore, volumetry can be used as a tool to guide diagnosis and discriminate between epilepsy types, which will be otherwise challenging if done solely by clinical examination [48]. In addition to manual segmentation, the improvement of MRI technology has also led to the development of more specific protocols for automatic hippocampal segmentation [49,51,52]. Automatic segmentation in epilepsy patients has a well-established validity and reliability as there is no significant difference when compared with expert raters [49].

Apart from volumetric MRI, other techniques to assess functionality and epileptogenic source localization are electroencephalography (EEG), functional MRI (fMRI), positron emission tomography (PET), and single-photon emission tomography (SPECT). For example, by itself, the use of fMRI allows for the assessment of the impact of epileptic activity over physiological tasks of the brain such as language, consequently allowing clinicians to predict possible post-surgical deficits [53,54]. However, when combining EEG and fMRI, a more precise identification of the epileptogenic zone can be achieved thanks to combining the hemodynamic and electrographic information, by tracking the seizure-related electrical activity in the spatial resolution of fMRI [55,56]. As a matter of fact, all of the previously mentioned imaging techniques can be used to determine the location of the epileptogenic foci with a high degree of certainty when assessed together (multimodality imaging), and multiple institutional protocols report using them to determine which patients are candidates for epilepsy surgery [57,58]. The development of these multimodality imaging protocols for the preoperative assessment of epilepsy surgery candidates has decreased the risks of the procedure and reduced the surgical footprint in the brain [57]. To be precise, this three-dimensional multimodal mapping is composed of CT, T1-weighted MRI, and CT angiography or resonance angiography, which may be combined with SPECT, PET, and fMRI. This allows for the planning of optimum different trajectories for electrode implanting, allowing a proper identification of the epileptogenic zone for a subsequent resection and considering brain structural lesions, crucial white matter tracts observed via tractography, location of eloquent cortex by fMRI, location of major vessels, and any areas related to a previous brain surgery, such as burr holes or craniotomy [57]. However, it is important to highlight that the techniques used in multimodality imaging are not all used to attain an exact anatomical localization but rather to predict and avoid lesions to relevant structures during surgery by means of a functional assessment, such as hemodynamic or metabolic changes [55].

It is interesting to note that, most of these imaging techniques have been used to assess the human PC but not to the same extent as other mesial temporal structures [16]. Few human studies have focused on evaluating the PC’s anatomy, localization, and volumetry [14,17,18,19]. In the oldest study, the anatomical limits of the PC on MRI images were determined by a histological analysis of 23 autopsies from a control group [17]. Their results demonstrated that TLE patients actually had a reduced PC volume ipsilateral to the side of epilepsy (*p* < 0.001) and that 46% of the patients with epilepsy had a reduction of more than 20% of their PC volume compared to the control group [17]. However, the volumetric analysis they performed did not include the frontal part of the PC, an area that has important connections within the human epileptic network [15,16]. The proportion of the PC resected in patients with seizure freedom during the surveillance time was larger than in patients with no seizure freedom (median, 83% [IQR, 64–91%] vs. 52% [IQR, 32–70%]; *p* < 0.001) [14]. Even though volumetric imaging techniques were applied in both studies to assess the human PC, the method used by each one was different, including a stereological procedure (Cavalieri method) and VBM [14,17]. Another important methodological caveat to consider is that the most recent study created a segmentation protocol based on the results from the previous study since the latter tailored the segmentation protocol to the histological findings of conducted autopsies [14,17]. It is obvious that any confounding factors affecting the segmentation protocol will propagate from one study to the other. Hence, studying the human PC is feasible, and more volumetry-based imaging investigations should be performed using different methodologies and protocols to obtain results that could be compared with the existing literature or used to validate their results.

Other authors, rather than analyzing the anatomical structure of the PC, focused on functionality by using studies such as EEG and nuclear medicine (PET, SPECT). Flanagan and collaborators were able to identify that the ipsilateral PC was a common node that became activated in patients with focal epilepsy, using a combination of EEG and fMRI [59]. This study replicated successfully the results obtained by Laufs and collaborators, who used a different combination that involved fMRI and PET [60].

These imaging techniques are quite useful as a diagnostic and prognostic tool. However, another important application of imaging in DR-TLE patients is its utility in creating a tailored surgical plan for those who fulfill certain criteria [58,61]. The combination of these tools has not only allowed for the identification of the source of epileptic seizures but also for the parcellation of specific structures like the PC to maximize its resection area [14,62,63].

## 4. The Role of Surgery in Temporal Lobe Epilepsy and the Influence of Piriform Cortex Response—Future Directions

The role of surgery in TLE patients who are resistant to pharmacological treatment is clearly established [8,9,10,64,65,66]. The most commonly used surgical techniques for this purpose are anterior temporal lobectomy (ATL) and selective amygdalohippocampectomy (SAH), both open surgeries [10]. In ATL, the amygdala and the hippocampus are surgically removed, along with 4–6 cm of the anterior portion of the temporal lobe, whereas in SAH, the temporal lobe neocortex is preserved [10]. As open surgeries, ATL and SAH are associated with important complications such as hemiparesis, temporal atrophy, wound infection, and cerebrospinal fluid leakage [67,68]. Furthermore, when looking at seizure freedom, no significant difference has been reported between these two techniques; however, available studies have used different time intervals after surgery to assess seizure freedom, and some have used different scales to determine such freedom, which makes the comparison between techniques a strenuous endeavor [10,69]. Furthermore, not all patients achieve seizure freedom; 20–30% of patients have persistent seizures after the first surgery and 53% after the second one [12,13].

Several reasons could explain these results. First, an insufficient resection of mesial structures could affect seizure outcome, since the structures that are not resected could still act as epileptogenic foci. Second, additional epileptogenic foci could exist in the contralateral temporal lobe or in extratemporal structures that were not properly identified before surgery [14,70]. When looking at incomplete resection of mesial structures, a study published in 2019 proposed that the lack of seizure freedom after open surgeries could be due to incomplete resection of the PC in TLE surgical candidates, rather than other mesial structures [14]. Certainly, they showed that patients in the seizure-free group had a higher volume of PC resected, pointing towards the importance of considering PC extirpation in the surgical plan for TLE-DR patients [14]. As thoroughly discussed before, it is logical to consider the PC in the surgical resection plan of TLE patients, given its myriad of connections to different parts of the brain and its epileptogenic potential. However, of the numerous surgical techniques that have been used to treat TLE (some minimally invasive), none of them consider PC resection as a part of the surgical plan. A summary of the principal surgical interventions used in TLE can be found in Table 1.

Minimally invasive surgical options such as radiosurgery, laser, or radiofrequency ablation are more desirable than the classic options (ATL or SAH), as they have a smaller surgical footprint in the brain. However, caution should be exercised as more robust evidence is required to fully recommend these procedures. For instance, stereotactic radiosurgery (SRS), which uses different forms of radiation and imaging techniques to guide the energy toward a target with a precision that could reach 0.3 mm, could be an important candidate for minimally invasive TLE surgery [67,74,75]. The seizure freedom rate of SRS has been reported to be similar to or lower than that of SAH, and it is associated with a risk of a temporary increase in seizure frequency [67]. Perhaps, the lack of benefit of SRS could be that the PC is not being targeted. Considering that the entire PC has approximately a length of 1.96 cm and a volume of 280 ± 27 mm3 [17], SRS, with its 0.3 mm precision, could easily target the PC in TLE patients and potentially result in better seizure freedom outcomes. To achieve this, proper protocols need to be established and validated in animal and human studies and be tested further through randomized clinical trials. Another alternative could be stereoelectroencephalography-guided radiofrequency thermocoagulation (SEEG-guided RF-TC), which uses electroencephalography to localize the epileptogenic foci zone and radiofrequency to produce thermocoagulation of the area [71,76]. However, there is not adequate evidence for this technique to have a proper therapeutic indication or be part of a diagnostic algorithm in TLE patients [77]. Finally, in contrast, laser interstitial thermal therapy (LITT) has proven to be a less invasive and effective procedure to treat TLE patients [67,72,73,78]. Human LITT has already been used in other kinds of procedures such as gliomas and brain metastases [67], and it involves the use of a stereotactic laser that enters through a tiny burr hole in the skull and performs thermal ablation with the guidance of real-time MRI and/or EEG monitoring [67,72]. However, the main targets of LITT in the treatment of TLE are the hippocampus, subiculum, amygdala, and uncus [67,72,73,78]; the PC is not targeted, despite its thorough and strong connections with the areas that are being targeted (Figure 4). In general, the PC is being neglected in most, if not all, of the classic and minimally invasive surgical techniques to treat TLE, perhaps explaining the lack of seizure freedom after surgery and the differences in outcomes between the classical techniques and the minimally invasive ones. In the classic techniques, there is a higher likelihood of removing the PC along with the other mesial temporal structures, whereas, in the minimally invasive techniques, the targeted area is much smaller, and the PC can be completely neglected.

When looking at seizure freedom as the main outcome of the aforementioned techniques, traditional surgical techniques have shown a better seizure freedom rate than minimally invasive procedures such as radiofrequency ablation or LITT [69]. ATL and SAH reach a seizure freedom rate between 65–70%, that value varies from 57 to 59% in LITT and from 21 to 44% in radiofrequency ablation techniques (RFA) as SEEG-guided RF-TC, considering a follow-up time of at least 6 months [69,78,79]. This difference becomes more statistically significant when the follow-up time is longer [69]. The non-statistically significant percentage difference between LITT and RFA techniques could be due to the capacity to repeat the LITT procedure and create overlapping thermal lesions [78]. Even with better outcomes, traditional techniques have a higher risk of complications, with 18 to 31% of patients affected, while LITT has a complication rate of 17%, and RFA a rate of 18.5% [67,69]. The most common complications with LITT are visual field defects, due to lesions in the optic radiation, such as homonymous hemianopsia or quadrantinopias; intracranial hemorrhages and subdural hematomas can also occur [67,69,72]. RFA complications include neurologic deficits, hemorrhage, and edema [78]. Intracranial hemorrhage risk is similar for minimally invasive and open surgeries, but in the latter, the risk of infection, stroke, and neuropsychological deficits is higher. Additionally, a shorter length of stay has been noted with minimally invasive procedures [69]. The outcome ratio for LITT and RFA presents a lot of heterogeneity; this could be due to the different TLE etiologies [78]. To select a surgical method, the etiology of TLE and the consideration of advantages and risks of every one of them should be taken into account, along with the fact that minimally invasive techniques have not been practiced for long, and their long-term outcomes cannot yet be directly compared with the ones from classic surgeries [69,78]. However, minimally invasive procedures could potentially become an important technique for TLE patients if they can properly target the correct epileptogenic structures; further studies, especially randomized controlled trials, are required to validate these hypotheses.

As thoroughly discussed throughout our paper, we believe that one of those targets should be the PC; therefore, more studies are required to assess the PC as a potential surgical target, both for traditional and minimally invasive techniques. Therefore, to establish the PC as a viable therapeutic target in TLE, we propose the subsequent roadmap for forthcoming studies: (1) Initial research must concentrate on enhancing animal models of TLE that accurately replicate human PC anatomy and performance. These models can assess the safety and efficacy of PC-targeted therapies, such as laser interstitial thermal therapy (LITT) and stereoelectroencephalography-guided radiofrequency thermocoagulation (SEEG-guided RF-TC). Multimodal imaging techniques, including diffusion tensor imaging (DTI) and functional MRI (fMRI), must be refined to evaluate the connectivity of the PC and inform therapies. (2) Small-scale studies in meticulously chosen patient populations should be conducted to evaluate the feasibility and safety of PC targeting. Outcomes must encompass seizure frequency, cognitive impacts, and procedure problems. (3) Collaboration among epilepsy centers will be essential for executing randomized controlled trials (RCTs) with varied patient populations. These studies ought to compare PC-targeted therapies with conventional and other minimally invasive methods, employing standardized imaging methodologies and outcome metrics. (4) Sophisticated imaging methodologies, including connectomes and real-time MRI guidance, must be investigated to enhance surgical accuracy and reduce complications. Protocols integrating imaging, electrophysiological, and computational modeling may improve our comprehension of the PC’s function in TLE and promote safer navigation within the operating room. (5) Research must incorporate prolonged follow-up durations to assess the sustainability of seizure management, cognitive results, and quality of life following surgery. This data will be crucial for establishing PC-oriented therapies as a regular choice in TLE care.

However, the anatomical location of the PC, in proximity to vital structures such as the lenticulostriate arteries, presents considerable surgical difficulties. Complications may encompass vascular injuries resulting in cerebral bleeding, cognitive impairments, or inadvertent harm to adjacent brain areas associated with olfaction and cognition [14,17,18]. Minimally invasive procedures, however safer, are not devoid of risk. Laser interstitial thermal therapy (LITT) may induce visual field impairments from damage to the optic radiation, whereas SEEG-guided RF-TC poses a risk of thermal injury to adjacent structures [14,17,18]. Therefore, minimally invasive surgical procedures aimed at the PC should be undertaken with prudence due to insufficient evidence regarding their safety and effectiveness. Ethical considerations encompass acquiring informed consent for experimental procedures, guaranteeing fair access to innovative treatments, and weighing the surgical risks against prospective benefits. Moreover, the invasiveness of certain procedures may be unwarranted for individuals experiencing moderate or occasional seizures, prompting worries regarding over-treatment. Nonetheless, the advancement of sophisticated imaging procedures may alleviate these dangers by facilitating accurate targeting and circumventing essential structures. Protocols that integrate imaging and intraoperative monitoring may significantly improve the safety of these procedures for drug-resistant TLE patients.

## 5. Conclusions

This review discusses all the factors that make the piriform cortex (PC) a key node in temporal lobe epilepsy (TLE) genesis and treatment outcomes. Its location and neural connections with epileptogenic regions make it a possible seizure initiator and perpetuator. This region is poorly described in humans, but multimodality imaging has helped identify it and enable real-time image guidance in the operating room during epilepsy surgery. Despite its importance in TLE epileptogenesis, few studies have examined this region as a therapeutic target. The etiology of TLE and the benefits and disadvantages of each surgery must be considered when a patient is a candidate for epilepsy surgery. Minimally invasive procedures are promising, but their long-term results are untested. However, targeting epileptogenic structures could make these approaches useful in TLE treatment. The PC is a promising surgical target, although its proximity to the lenticulostriate arteries makes minimally invasive treatments difficult. Future research should create multimodal imaging procedures to reduce hazards and validate the PC as a primary target in traditional and less invasive TLE surgeries.

## Figures and Tables

**Table 1:** Positive and negative aspects of different surgical techniques used to treat drug-resistant temporal lobe epilepsy.

Surgical Techniques	ATL	SAH	SRS	SEEG-Guided RF-TC	LITT
Positive aspects	– Commonly used technique for drug-resistant TLE treatment [10]	– Commonly used technique for drug-resistant TLE treatment [10] – Temporal lobe neocortex preservation [10]	– Less invasive technique, decreased wound infection risk [67] – Shorter recovery time [67]	– Less invasive technique, decreased wound infection risk [71] – Allows to target a specific area thanks to real-time guidance [71] – Shorter recovery time [71]	– Less invasive technique, decreased wound infection risk [67,72] – Has already been used in other types of surgery [67] – MRI real-time monitoring [67,72] – Better seizure-free outcomes [72,73] – Preservation of neurocognitive functions [67,72] – Shorter recovery time [67]
Negative aspects	– Invasive, higher risk of wound infection – Greater risk of neocortical lesion [10] – Longer recovery time [67,68] – Seizure recurrence because of residual mesial structures [10] – Other complications such as temporal atrophy and hemiparesis [67,68]	– Invasive, higher risk of wound infection [67,68] – Longer recovery time [67,68] – Seizure recurrence in a large proportion of patients [12,13] – Other complications such as temporal atrophy and hemiparesis [67,68]	– Seizure freedom rate similar to SAH [67] – Temporary risk of higher seizure frequency [67]	– Less effective than classical techniques: ATL and SAH [71]	– Risk of damaging relevant structures, most commonly optic radiations [67,72]

ATL: anterior temporal lobectomy; SAH: selective amygdalohippocampectomy; SRS: stereotactic radiosurgery; SEEG-Guided RF-TC: stereoelectroencephalography-guided radiofrequency thermocoagulation; LITT: laser interstitial thermal therapy.

## Data Availability

The original contributions presented in the study are included in the article; further inquiries can be directed to the corresponding author.
